# Association of dietary inflammatory index with gynecological cancers in NHANES 2011–2018

**DOI:** 10.3389/fnut.2025.1560987

**Published:** 2025-05-12

**Authors:** Chen Chen, Mengyu Zheng, Xing Dong, Pei Zhang, Zhuo Bao, Yushan Cao, Yixuan Liu, Jinxiang Yan, Yongzhen Guo, Xianxu Zeng

**Affiliations:** ^1^Department of Pathology, The Third Affiliated Hospital of Zhengzhou University, Zhengzhou, China; ^2^Zhengzhou Key Laboratory of Gynecological Disease’s Early Diagnosis, Zhengzhou, China

**Keywords:** dietary inflammatory index (DII), gynecological cancers (GC), NHANES (National Health and Nutrition Examination Survey), RCS, smoking

## Abstract

**Objective:**

This study aimed to analyze the association between the Dietary Inflammatory Index (DII) and the risk of gynecological cancers using data collected from the National Health and Nutrition Examination Survey (NHANES) between 2011 and 2018.

**Methods:**

The data for this study were obtained from NHANES, conducted between 2011 and 2018, and included a total of 8,380 women. To examine the association between the Dietary Inflammatory Index and gynecological cancers, weighted multivariable logistic regression analyses were performed, using DII both as a continuous variable and as a categorical variable divided into tertiles. Subgroup analyses stratified by DII and gynecological cancer characteristics were conducted to further explore this association. Additionally, restricted cubic spline (RCS) analysis was applied to evaluate potential non-linear relationships between DII and gynecological cancer risk.

**Results:**

Among the 8,380 women included in the analysis, the mean age was 47.02 (SD: 16.91) years, and 196 participants self-reported a diagnosis of gynecological cancer. In fully adjusted models, DII was significantly positively associated with the prevalence of gynecological cancer, whether analyzed as a continuous variable (OR = 1.15, 95% CI: 1.00–1.33, *p* = 0.046) or as a categorical variable (highest tertile compared to the lowest tertile: OR = 2.14, 95% CI: 1.14–4.04, *p* = 0.021, p for trend = 0.021). Restricted cubic spline analysis confirmed a linear relationship between DII and gynecological cancer risk (p for non-linear association = 0.1984). Subgroup analyses revealed a significant interaction effect with smoking status (p for interaction = 0.037).

**Conclusion:**

Our findings suggest that higher DII scores are positively associated with an increased risk of gynecological cancer. These results contribute to the existing literature and underscore the need for further validation through larger prospective cohort studies.

## Introduction

Gynecological cancers (GC), including cervical cancer (CC), endometrial cancer (EC), and ovarian cancer (OC), represent a growing global public health challenge with increasing incidence and mortality rates (1, 2).

Globally, cervical cancer is the fourth most common cancer among women. According to data from the World Health Organization, in 2022, there were 660,000 new cases of cervical cancer worldwide, with 350,000 deaths (3). In parallel, the incidence and mortality trends of endometrial cancer are also a cause for concern. In 2023, the United States reported 66,200 new cases of endometrial cancer, with 13,030 deaths. It is projected that the incidence of endometrial cancer will increase by 40-50% by 2030 (4). Ovarian cancer, the eighth most common cancer among women, is the most lethal gynecological malignancy. In 2023, approximately 19,710 new cases of ovarian cancer were diagnosed in the United States (5, 6). Despite its relatively lower incidence, ovarian cancer is highly lethal due to its asymptomatic nature and lack of effective early detection, resulting in late-stage diagnoses and poor prognosis.

Collectively, these gynecological cancers pose a substantial burden on healthcare systems and significantly diminish the quality of life for affected women. The rising trends in incidence, mortality, and disability-adjusted life years (DALYs) underscore the urgent need to strengthen preventive measures, early screening programs, and treatment strategies (7, 8).

Primary prevention is widely regarded as one of the most effective and cost-efficient strategies to fight cancer. Current evidence suggests that between a third and a half of all cancers are preventable (9). The World Health Organization (WHO) Global Status Report on Non-communicable Diseases (NCDs) identifies several key risk factors for cancer, including unhealthy diets, tobacco use, alcohol consumption, and physical inactivity (10). Among these, diet has emerged as a critical factor. Parkin and colleagues identified diet as the second most significant modifiable risk factor for cancer, following tobacco use (11). Further research suggests that if modifiable risk factors, such as tobacco use and high salt intake, were reduced to optimal levels or eliminated, approximately 40% of cancer cases in women could be prevented (12).

In recent years, a growing body of evidence has underscored the significant role of dietary patterns and nutritional quality in modulating cancer risk, particularly in the prevention of gynecological cancers. Dietary modifications and improvements in nutritional quality have become increasingly crucial components of prevention strategies in this field. Dietary Inflammatory Index is a comprehensive tool designed to evaluate the inflammatory potential of diets and has been widely utilized in studies investigating the associations between diet and disease. Developed by Shivappa et al., the DII is based on a systematic review of literature and integrates the pro-inflammatory and anti-inflammatory properties of 45 dietary components (13). Unlike conventional methods that primarily focus on single nutrients or specific foods, the DII assesses the overall inflammatory potential of the entire diet. Each dietary component is assigned a score based on its validated inflammatory properties through extensive research, with higher DII scores indicating stronger pro-inflammatory potential (14–16). This tool is useful for evaluating the dietary inflammatory levels of individuals or populations with complete dietary data. Previous studies have reported that higher DII scores are associated with increased risks of metabolic syndrome (17), cardiovascular disease (18), cancer (19, 20), and sarcopenia (21, 22). These findings emphasize the importance of exploring the role of DII in disease treatment and prevention.

Despite the growing number of studies examining the relationship between DII and cancer risk, research specifically focusing on gynecological cancers remains limited. Most existing studies have addressed single cancer types or isolated risk factors, with few exploring the comprehensive relationship between dietary inflammatory potential and gynecological cancers. Therefore, further investigations are needed to elucidate the potential association between DII and gynecological cancers, providing evidence to support the development of targeted dietary interventions.

In this study, we conducted a cross-sectional analysis using data from the 2011–2018 National Health and Nutrition Examination Survey to explore the associations between the Dietary Inflammatory Index and gynecological cancers, including cervical cancer, endometrial cancer, and ovarian cancer. Our study aims to address the gaps in the existing literature and provide novel insights to inform the prevention and management of gynecological cancers.

## Materials and methods

### Study population

NHANES, a substantial and nationally inclusive survey, is crafted to evaluate the health and nutritional condition of the American population. This survey is conducted by the National Center for Health Statistics at the United States. Centers for Disease Control and Prevention (23). Since 1999, the NHANES has conducted cross-sectional surveys, releasing new data every 2 years. This study, which utilized the data from NHANES (2011–2018), involved a total of 22,616 participants.

**FIGURE 1:**
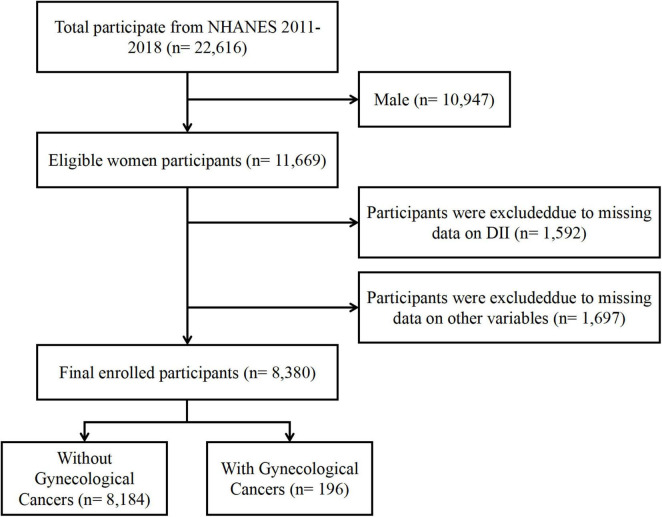
Flowchart of the study population. NHANES, National Health and Nutrition Examination Survey; DII, Dietary Inflammatory Index.

We excluded men (*N* = 10,947), women with no DII data (*N* = 1,592) and those with no covariates data (*N* = 1,697), resulting in 8,380 subjects being included in our study (Figure 1). Among these participants, 196 self-reported a history of gynecologic cancers, including 95 with cervical cancer, 63 with endometrial cancer, and 38 with ovarian cancer. The NHANES protocol received approval from the National Center for Health Statistics (NCHS) Research Ethics Review Board, and informed consent was obtained from all participants prior to inclusion in the study.

### Dietary inflammation index calculation

Dietary data for this study were primarily collected by the Nutrition Methods Working Group through face-to-face and telephone interviews at Mobile Examination Centers (MEC) using two 24-h dietary recall questionnaires. Oversight and implementation of the dietary data collection methodology, database maintenance, and data review were managed by the Food Surveys Research Group within the US Department of Agriculture. Participants who were absent from the dietary recall interview were excluded.

In this study, we calculated the DII using the “dietaryindex” R package developed by Zhan et al., which has been validated against standard DII scoring algorithms (24, 25). From the NHANES 2011-2018 survey cycle, 27 dietary components were included in our analysis. Previous studies have confirmed the stable predictive validity of DII when utilizing this set of dietary components (14, 26). Detailed specifications of these 27 dietary components are provided in Supplementary Table 1. Six different inflammatory markers were used to assess various levels of inflammation. Components that significantly increased the levels of interleukins (IL)-1β, IL-6, C-reactive protein (CRP), tumor necrosis factor (TNF)-α, or significantly decreased the levels of IL-4 and IL-10 were scored as “ +1”. Conversely, components that decreased the levels of IL-1β, IL-6, CRP, and TNF-α, or increased the levels of IL-4 and IL-10 were scored as “–1”. If a dietary component did not alter the levels of inflammatory markers, it was deemed to have no inflammatory properties and was scored as “0”. In the overall inflammatory index, positive scores indicate pro-inflammatory potential, whereas negative scores indicate anti-inflammatory potential.

The formula for calculating DII is: (Daily intake of a dietary component – Global daily average intake of the component)/Standard deviation of the global daily intake of the component * Overall inflammatory effect score of the dietary component.

Finally, the overall DII score for each individual was calculated as the sum of the DII scores for each specific food parameter. A higher DII score reflects greater dietary inflammatory potential (27). In addition, beyond analyzing DII as a continuous variable, we categorized DII into tertiles for further analysis. Participants in the highest tertile of DII were classified as consuming a pro-inflammatory diet in this study.

### Diagnosis of cancer

Data on cancer diagnoses were obtained from a structured questionnaire. Participants were asked if a doctor or other health professional had ever informed them of a cancer or malignancy diagnosis (MCQ-220). Participants who answered affirmatively were identified as cancer patients and were subsequently prompted to answer MCQ-230A, where they were further asked about the specific type of cancer they had. In MCQ-230A, code 15 indicates cervical cancer, code 28 indicates ovarian cancer, and code 38 indicates endometrial cancer.

### Covariates

Based on clinical practice, previous literature, and data available in the NHANES database (28–32), we selected the following covariates to control for potential confounding bias in this study: age, race, marital status, education level, poverty income ratio (PIR), exercise status, smoking status, alcohol consumption, and history of hypertension and diabetes. Additionally, considering the close association between the occurrence of gynecological cancers and hormone levels, we also included behavioral factors that could influence hormone levels, such as the use of hormonal treatments and birth control pills, as covariates.

Racial classification included the following groups: “Mexican American,” “Other Hispanic,” “Non-Hispanic White,” “Non-Hispanic Black,” and “Other/more than one race.” Marital status was categorized into “Married,” “Living with partner,” and “Alone.” Educational attainment was categorized into two levels: “Less than high school,” and “High school or above.” Smoking status was classified as either “Yes” or “No” based on self-reported consumption of at least 100 cigarettes over the individual’s lifetime. Alcohol use was similarly categorized as “Yes” or “No” based on self-reported consumption of at least 12 alcoholic drinks per year. Exercise activity was defined as participation in any vigorous—intensity exercise or recreational activity lasting at least 10 continuous min per week that caused substantial increases in breathing or heart rate, such as running or playing basketball. Participants were considered to have hypertension if they had been told by their doctors that they had hypertension, or systolic blood pressure was 140 mmHg or greater, or diastolic blood pressure was 90 mmHg or greater. Presence of diabetes mellitus was determined if participants were told they had diabetes mellitus, or were taking glucose—lowering drugs, or glycosylated hemoglobin (%) was 6.5% or greater during the NHANES test.

### Statistical analysis

For the statistical analysis of this study, NHANES took survey weights into account. Continuous variables were presented as weighted means (± standard error, SE), and categorical variables were presented as weighted counts (weighted percentages).

Following the Strengthening the Reporting of Observational Studies in Epidemiology (STROBE) guidelines (33), two multivariate regression models were constructed. In model 0, no covariates were adjusted. In model 1, age, race, marital status, and education level were adjusted. Model 2 was adjusted for age, race, marital status, education level, PIR, exercise status, smoking status, alcohol consumption, BMI, hypertension, diabetes, use of female hormones, and use of birth control pills.

To assess its robustness, the continuous variable DII was categorized into tertiles for sensitivity analysis.

We conducted a further evaluation of the differences in the risk of gynecological cancers among the tertile groups of dietary inflammatory index, using the T1 group as the reference. Additionally, we employed restricted cubic spline curves derived from Model 2 to investigate potential non-linear relationships between the dietary inflammatory index and gynecological cancers.

Finally, we conducted interaction and stratified analyses based on age, race, marital status, education level, PIR, exercise status, BMI, smoking status, alcohol consumption, hypertension, diabetes, use of female hormones, and use of contraceptives. The statistical software packages R^1^ and Empower Stats^2^ were used for analysis. *P* < 0.05 was considered statistically significant.

## Results

### Baseline characteristics

Baseline characteristics of the participants are shown in Table 1. A total of 8,380 female participants were included in the study, with a mean age of 47.02 (SD: 16.91) years. Among them, 196 participants were identified as having gynecological cancers, while the remaining 8,184 participants did not. Detailed information on cancer types and their distribution is provided in Supplementary Table 2.

**TABLE 1:** Baseline characteristics of participant.

Characteristic	Total (*n* = 8,380)	Non-GC (*n* = 8,184)	GC (*n* = 196)	*P*-value
**Age [years, mean (SD)]**	47.02 ± (16.91)	46.82 ± (16.94)	54.20 ± (14.03)	<0.001
**Race (*N*,%)**				<0.001
Mexican American	1,164 (8.14%)	1,134 (8.17%)	30 (6.97%)	
Other Hispanic	919 (6.24%)	901 (6.28%)	18 (4.54%)	
Non-Hispanic White	3,067 (65.07%)	2,959 (64.69%)	108 (78.61%)	
Non-Hispanic Black	1,951 (12.06%)	1,928 (12.30%)	23 (3.86%)	
Other race—including multi-racial	1,279 (8.49%)	1,262 (8.56%)	17 (6.02%)	
**Marital status (*N*,%)**				0.593
Married	3,822 (51.33%)	3,745 (51.37%)	77 (50.03%)	
Living with partner	711 (8.58%)	694 (8.63%)	17 (6.91%)	
Alone	3,847 (40.09%)	3,745 (40.00%)	102 (43.06%)	
**Education level (*N*,%)**				0.246
Less than high school	1,665 (12.66%)	1,612 (12.58%)	53 (15.45%)	
High school or above	6,715 (87.34%)	6,572 (87.42%)	143 (84.55%)	
**PIR [%, mean (SD)]**	2.87 ± (1.64)	2.87 ± (1.64)	2.64 ± (1.61)	0.214
**Exercise status (*N*,%)**				0.177
Yes	3,589 (47.85%)	3,514 (48.06%)	74 (40.68%)	
No	4,791 (52.15%)	4,670 (51.94%)	122 (59.32%)	
**Smoking (*N*,%)**				<0.001
Yes	2,816 (36.65%)	2,709 (36.11%)	107 (55.96%)	
No	5,564 (63.35%)	5,475 (63.89%)	89 (44.04%)	
**Drinks (*N*,%)**				0.646
Yes	5,179 (69.60%)	5,052 (69.54%)	127 (71.76%)	
No	3,201 (30.40%)	3,132 (30.46%)	69 (28.24%)	
**Hypertension (*N*,%)**				<0.001
Yes	3,093 (34.19%)	2,990 (32.63%)	103 (47.13%)	
No	5,287 (65.81%)	5,194 (67.37%)	93 (52.87%)	
**Diabetes (*N*,%)**				0.361
Yes	1,314 (11.87%)	1,278 (11.78%)	36 (14.97%)	
No	7,066 (88.13%)	6,906 (88.22%)	160 (85.03%)	
**Female hormones (*N*,%)**				<0.001
Yes	1,322 (19.08%)	1,246 (18.36%)	76 (44.40%)	
No	7,058 (80.92%)	6,938 (81.64%)	120 (55.60%)	
**Birth control pills (*N*,%)**				0.388
Yes	5,602 (74.21%)	5,468 (74.30%)	134 (71.18%)	
No	2,778 (25.79%)	2,716 (25.70%)	62 (28.82%)	
**BMI [kg/m^2^, mean (SD)]**	29.59 ± (7.71)	29.56 ± (7.72)	30.48 ± (7.63)	0.141
**DII [mean (SD)]**	1.21 ± (1.96)	1.19 ± (1.96)	1.71 ± (1.92)	0.021
**DII Tertile (*N*,%)**				0.037
T1	2,794 (35.31%)	2,750 (35.68%)	44 (22.28%)	
T2	2,793 (33.73%)	2,725 (33.58%)	68 (39.26%)	
T3	2,793 (30.96%)	2,709 (30.75%)	84 (38.46%)	

For continuous variables: *P*-value was by survey-weighted linear regression. For categorical variables: *P*-value was by survey-weighted Chi-square test. GC, Gynecological Cancers (including cervical cancer, endometrial cancer, and ovarian cancer); PIR, Ratio of Family Income to Poverty; BMI, Body Mass Index; DII, Dietary Inflammatory Index; T, Tertile; SD, Standard Deviation.

Statistically significant differences were observed between the two groups with respect to age, race, smoking status, hypertension, and the use of exogenous female hormones (*p* < 0.05). In contrast, no significant differences were detected in marital status, educational attainment, alcohol consumption, diabetes prevalence, use of oral contraceptives, or body mass index (*p* > 0.05). Furthermore, patients diagnosed with gynecological cancer exhibited significantly higher Dietary Inflammatory Index levels compared to healthy participants (weighted mean DII, 1.71 vs. 1.19, *p* = 0.021).

### Association between DII and gynecological cancers

The results of both univariate and multivariate logistic regression models highlight the importance of dietary pattern interventions in primary cancer prevention. Table 2 presents the results of the logistic regression analysis examining the relationship between the Dietary Inflammatory Index and gynecological cancers. In the non-adjusted model (Model 0), DII demonstrated a positive association with gynecological cancers, both as a continuous variable (OR = 1.16, 95% CI: 1.01–1.32, *p* = 0.033) and as a categorical variable, with the highest tertile compared to the lowest (OR = 2.13, 95% CI: 1.15–3.95, *p* = 0.017; p for trend = 0.017).

**TABLE 2:** Multi regression analysis of the association between DII and gynecological cancers.

Characteristic	Model 0	Model I	Model II
**Continuous DII**	1.16 (1.01, 1.32) 0.033	1.16 (1.01, 1.33) 0.037	1.15 (1.00, 1.33) 0.046
**DII tertile (*N*,%)**			
T1	Reference	Reference	Reference
T2	1.69 (1.02, 2.80) 0.041	1.68 (1.00, 2.82) 0.048	1.71 (1.01, 2.91) 0.048
T3	2.13 (1.15, 3.95) 0.017	2.14 (1.15, 4.00) 0.018	2.14 (1.14, 4.04) 0.021
**P for trend**	0.017	0.018	0.021

Data are presented as OR [95% confidence interval] *P*-value. Model 0, No covariate was adjusted. Model I, Age, race, marital status, and education level were adjusted. Model II: Age, race, marital status, education level, PIR, exercise status, smoking status, alcohol consumption, BMI, hypertension, diabetes, use of female hormones, and use of birth control pills were adjusted. OR, Odds Ratio; DII, Dietary Inflammation Index; T, Tertile; PIR, Ratio of Family Income to Poverty; BMI, Body Mass Index.

Similarly, results from the minimally adjusted model (Model 1) and the fully adjusted model (Model 2) followed a consistent trend. DII was significantly associated with an increased risk of gynecological cancers in Model 1 (continuous variable: OR = 1.16, 95% CI: 1.01–1.33, *p* = 0.037; tertile: OR = 2.14, 95% CI: 1.15–4.00, *p* = 0.018; p for trend = 0.018) and in Model 2 (continuous variable: OR = 1.15, 95% CI: 1.00–1.33, *p* = 0.046; tertile: OR = 2.14, 95% CI: 1.14–4.04, *p* = 0.021; p for trend = 0.021). The results of the multivariate regression models, consistent with univariate regression results, suggest that DII is positively associated with gynecological cancer, even after adjustment for potential confounders.

### Non-linear relationship

**FIGURE 2:**
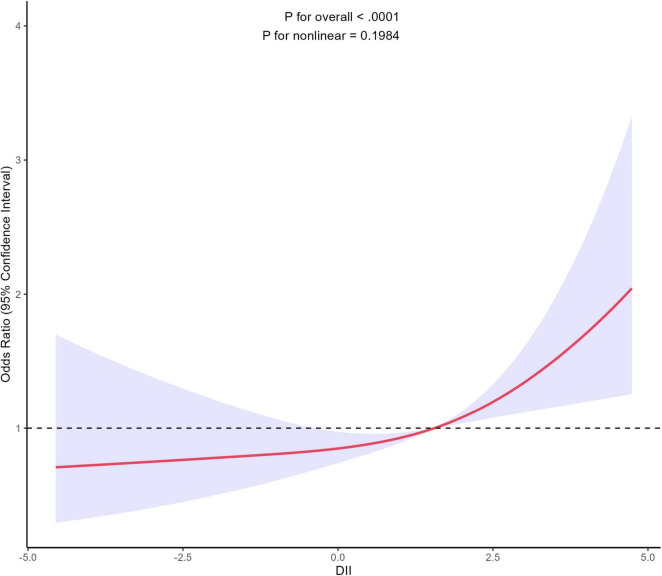
Association between DII and gynecological cancers using a restricted cubic spline regression model. Graphs show ORs for end according to DII adjusted for age, race, marital status, education level, PIR, exercise status, smoking status, alcohol consumption, BMI, hypertension, diabetes, use of female hormones, and use of birth control pills. Solid lines indicate ORs, and shadow shape indicate 95% CIs. OR, odds ratio; CI, confidence interval.

In order to assess the potential existence of a non-linear relationship between DII and gynecological cancers, we employed a 4-knot restricted cubic spline. The *p*-value for the non-linearity test was 0.1984, signifying the absence of a statistically significant non-linear correlation between DII and gynecological cancers. As shown in Figure 2, the curve illustrates a general increasing trend, suggesting a positive correlation between DII and the development of gynecological cancers. Additionally, Supplementary Figure 1 further illustrates the association between DII and gynecological cancers stratified by smoking status.

### Stratified analysis

**FIGURE 3:**
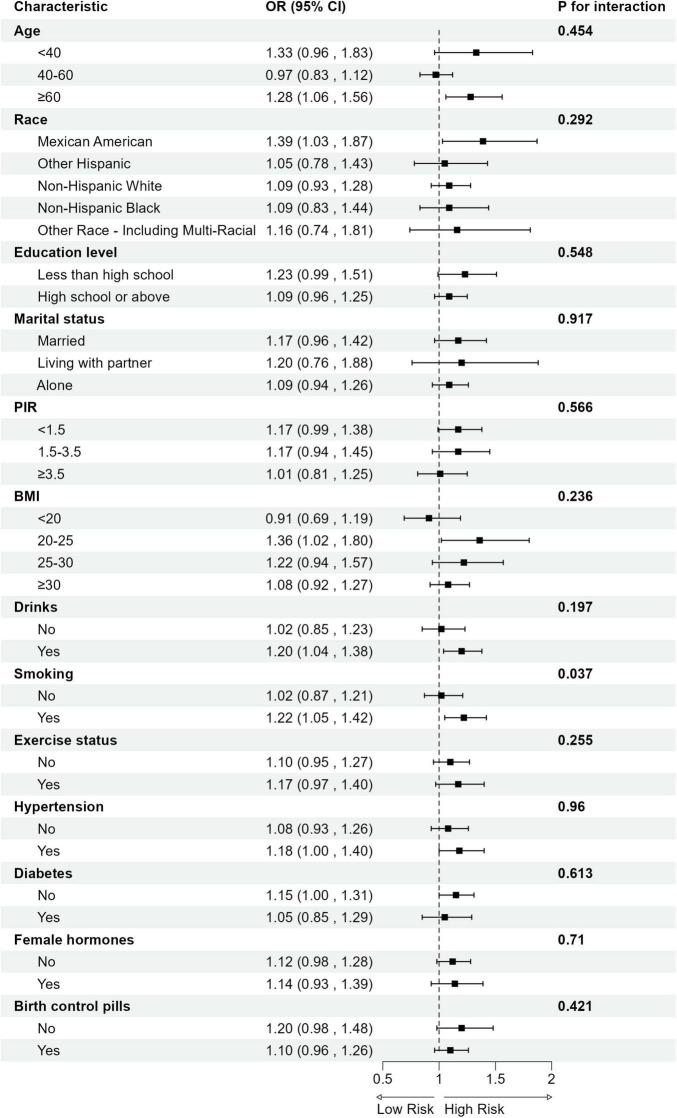
Subgroup analysis for the association between DII and gynecological cancers. OR, odds ratio; CI, confidence interval; PIR, Ratio of Family Income to Poverty; BMI, Body Mass Index.

Subgroup analysis revealed significant interaction effects of smoking status on the relationship between DII and gynecological cancers. No significant interaction was observed across other strata (Figure 3).

### The relationship between single nutrient and gynecological cancers

We further examined the association between individual dietary component intake and gynecological cancers, as presented in Supplementary Table 3. Dietary fiber and thiamin intake from the first 24-h recall showed negative associations with gynecological cancers. Conversely, a dose relationship between caffeine and gynecological cancers was found, which means too much caffeine may be positively correlated with gynecological cancers. In addition, Pearson’s and Spearman’s correlation coefficients were used to analyze the correlations among the concentrations of 27 dietary components (Supplementary Figures 2, 3 and Supplementary Table 4).

## Discussion

This study utilized data from the 2011–2018 National Health and Nutrition Examination Survey (NHANES) to construct the Dietary Inflammatory Index (DII) based on 27 dietary components associated with potential inflammatory effects. The primary objective was to evaluate the association between DII and the risk of gynecological cancers, including cervical, endometrial, and ovarian cancers. After adjusting for potential confounders, the results demonstrated that higher DII scores, reflecting a more pro-inflammatory dietary profile, were significantly associated with an increased risk of gynecological cancers. Specifically, each one-unit increase in DII score was linked to a 15% higher risk of developing gynecological cancers. Sensitivity and dose-response analyses further confirmed this association. Subgroup analyses revealed that smokers exhibited more pronounced adverse effects of elevated DII.

Our findings align with those of Romanos-Nanclares et al., who reported a positive association between the dietary inflammatory index and the risk of gynecological cancers (34–36). Furthermore, previous national studies have shown that overall dietary patterns vary across different racial and ethnic groups (37, 38), and both race and educational attainment have been demonstrated to influence the incidence of gynecological cancers (39, 40). In our study, the positive association between DII and the risk of gynecological cancers remained consistent across different racial/ethnic groups and levels of educational attainment.

Subgroup analysis further reveals that the association between elevated DII and increased risk of gynecological cancers is more pronounced among smokers (Smoking: Yes: OR = 1.22, 95% CI: 1.05–1.42 vs. No: OR = 1.02, 95% CI: 0.87–1.21; p for interaction = 0.04; FDR—corrected *p* = 0.44). Although the statistical significance of the interaction weakened after FDR correction, the observed trend indicates a potential interaction between smoking and pro-inflammatory diets in relation to gynecological cancer risk. Notably, this result is consistent with previous reports by Tran et al., which highlighted the pathogenic effects of smoking on gynecological cancers (41). Moreover, our findings provide new insights into the potential mechanisms underlying the interaction between dietary inflammation and smoking.

Smoking is a well-established pro-inflammatory and carcinogenic factor, with its effects likely driving cancer development through chronic inflammatory pathways. Studies have shown that polycyclic aromatic hydrocarbons and nitrosamines, key compounds in tobacco, directly induce DNA damage and genetic mutations, initiating tumorigenesis (42). However, smoking exerts its carcinogenic effects not only through direct genotoxic mechanisms but also by amplifying systemic chronic inflammation. Nicotine, a major bioactive compound in tobacco, activates nicotinic acetylcholine receptors (nAChRs), thereby promoting cell proliferation, inhibiting apoptosis, and inducing oxidative stress (43). These processes lead to the excessive release of pro-inflammatory cytokines, such as IL-6, TNF-α, and CRP, which establish a chronic inflammatory microenvironment (44). This inflammatory state serves as both a catalyst for cancer initiation and a foundation for amplifying the pro-inflammatory effects of a high-DII diet. The synergistic effect of smoking and dietary inflammation may involve epigenetic mechanisms. Smoking has been shown to induce widespread epigenetic alterations, marked by the hypermethylation of tumor suppressor genes (e.g., CDKN2A, BRCA1) and the hypomethylation of oncogenes, along with histone modifications and non-coding RNA dysregulation. These changes ultimately result in tumor suppressor gene silencing and the activation of oncogenic pathways (45–47). Simultaneous exposure to smoking and pro-inflammatory diets may result in a dual biological burden of chronic inflammation and epigenetic dysregulation, which may have a potential association with an increased risk of gynecological cancers.

This study presents critical implications for public health interventions. First, smoking has been established as an independent risk factor for gynecological cancers, making smoking cessation a cornerstone strategy in cancer prevention efforts. Second, optimizing dietary patterns among smokers has the potential to provide additional protective effects against gynecological cancers. Anti-inflammatory diets, characterized by a high intake of fruits, vegetables, and healthy fats, are rich sources of bioactive compounds, including polyphenols, phytoestrogens, and antioxidants. These bioactive compounds not only exhibit anti-inflammatory properties but may also exert protective effects by modulating estrogen levels and estrogen receptor signaling pathways. Chronic inflammation can enhance the bioactivity of estrogen and promote tumorigenesis through the activation of estrogen receptor pathways. However, phytoestrogens in anti-inflammatory diets may counteract the carcinogenic effects of endogenous estrogen by competitively binding to estrogen receptors. Additionally, these compounds may regulate epigenetic mechanisms, such as reversing abnormal DNA methylation and activating tumor suppressor genes, thus contributing to the prevention of gynecological cancers (48, 49).

Chronic inflammation is closely associated with cancer development (50–53), promoting tumor initiation and progression through the regulation of cytokine networks (54), oxidative stress responses (55), and immune evasion mechanisms (56). Diets with high inflammatory potential are typically rich in refined carbohydrates, saturated fats, and processed foods, while being deficient in anti-inflammatory components such as fruits, vegetables, and omega-3 fatty acids. This dietary pattern significantly increases the release of pro-inflammatory cytokines (57). Chronic inflammation not only serves as a major trigger for DNA damage but also activates multiple signaling pathways, such as NF-κB and STAT3, which induce the expression of cancer-related genes (58–62). This process disrupts the tissue microenvironment and exacerbates the risk of cancer development (63–65).

In gynecological cancers, the carcinogenic mechanisms of chronic inflammation exhibit certain specificities. For instance, cervical cancer is primarily associated with persistent infection by high-risk human papillomavirus (HPV). Pro-inflammatory diets may exacerbate this process by elevating levels of pro-inflammatory cytokines, which can further facilitate viral gene integration and expression, alter the immune microenvironment of host cells, and accelerate virus-related carcinogenesis (66). Endometrial cancer, on the other hand, is closely linked to obesity, insulin resistance, and elevated estrogen levels (67). The pro-inflammatory state in the body, often associated with a high BMI value, can disrupt the body’s hormonal levels, thereby increasing the risk of endometrial cancer (68–70). The etiology of ovarian cancer is more complex, and its exact mechanisms remain incompletely understood. Studies suggest that repetitive ovulation and the accompanying local repair-associated inflammatory responses may induce genetic mutations and promote malignant transformation (71). Pro-inflammatory diets may exacerbate localized inflammatory responses, alter immune cell activity, and modulate the levels of angiogenic factors in the ovarian microenvironment. Additionally, they may promote immune evasion by upregulating immune checkpoint molecules (e.g., PD-L1) and suppressing effector T cell function, thereby contributing to tumor progression.

Despite the differences in the specific mechanisms underlying each type of cancer, the overall impact of the DII appears consistent across these cancers. The findings of this study support the notion that populations with dietary habits characterized by high inflammatory potential have a greater likelihood of developing gynecological cancers.

This study has several limitations. The cross-sectional design of NHANES inherently limits causal inference due to the lack of temporal sequencing. Although the results demonstrate a positive association between higher DII scores and an increased risk of gynecological cancers, this design precludes establishing a cause-effect relationship. Furthermore, self-reported data is a common practice in epidemiological studies, though its accuracy may be limited by recall bias or respondent misinterpretation. These limitations can result in inaccuracies in both cancer diagnosis and dietary quality data, potentially impacting the reliability of the study’s findings.

## Conclusion

In conclusion, this study highlights a significant positive association between the Dietary Inflammatory Index and the risk of gynecological cancers. Public health strategies that promote anti-inflammatory dietary patterns rich in fruits, vegetables, whole grains, and healthy fats may play an important role in cancer prevention. Future research should further investigate the impact of anti-inflammatory diets among smokers to provide a scientific foundation for developing personalized prevention strategies for gynecological cancers. Additionally, prospective intervention trials are needed to determine whether anti-inflammatory dietary patterns can directly reduce the incidence of gynecological cancers by modulating inflammation markers, and to explore whether dietary habits change after a cancer diagnosis, thereby providing valuable evidence to support effective cancer prevention strategies.

## Data Availability

Publicly available datasets were analyzed in this study. This data can be found here: https://www.cdc.gov/nchs/nhanes/.

## Footnotes

1
http://www.R-project.org

2
http://www.empowerstats.com

## Supplementary material

Supplementary Figure 1 The association between DII and gynecological cancers stratified by smoking status.

Supplementary Figure 2 Pearson correlations among the 27 dietary components.

Supplementary Figure 3 Spearman correlations among the 27 dietary components.
