# Supplementary material for: Association of dietary inflammatory index with gynecological cancers in NHANES 2011–2018
Source: Front Nutr. 2025 May 12;12:1560987. doi: 10.3389/fnut.2025.1560987 (PMC12104050; doi:10.3389/fnut.2025.1560987)
Supplement: Supplementary file 1 [file Table_1.docx]

**Supplementary Table 1** Food parameters included to calculate the dietary inflammatory index.

| **Food parameters** |
| --- |
| Total fat, saturated fat, monounsaturated fatty acids, polyunsaturated fatty acids, *n*-3 Fatty acids, *n*-6 Fatty acids, energy, protein, carbohydrate, cholesterol, dietary fiber, caffeine, alcohol, folic acid, riboflavin, β-Carotene, vitamin A, thiamin, vitamin B6, niacin, vitamin B12, vitamin C, vitamin E, magnesium, iron, zinc, and selenium. |
