# Supplementary material for: Association of dietary inflammatory index with gynecological cancers in NHANES 2011–2018
Source: Front Nutr. 2025 May 12;12:1560987. doi: 10.3389/fnut.2025.1560987 (PMC12104050; doi:10.3389/fnut.2025.1560987)
Supplement: Supplementary file 2 [file Table_2.docx]

**Supplementary Table 2** Distribution of Gynecological cancer Types in the Study Population

| GC Type | Number of Cases (n) |
| --- | --- |
| Total GCs | 196 |
| Cervical cancer | 95 |
| Endometrial cancer | 63 |
| Ovarian cancer | 38 |
