# Supplementary material for: Association of dietary inflammatory index with gynecological cancers in NHANES 2011–2018
Source: Front Nutr. 2025 May 12;12:1560987. doi: 10.3389/fnut.2025.1560987 (PMC12104050; doi:10.3389/fnut.2025.1560987)
Supplement: Supplementary file 3 [file Table_3.docx]

| Supplementary Table 3. Associations between 27 dietary components and the risk of gynecological cancers from 2011-2018 NHANES analyzed by weighted logistic regression | | | |
| --- | --- | --- | --- |
| **Characteristic** | **Model 0** | **Model Ⅰ** | **Model Ⅱ** |
| Total fat (g) | 1.00 (1.00, 1.00) 0.200 | 1.00 (1.00, 1.00) 0.200 | 1.00 (1.00, 1.00) 0.200 |
| Saturated fat (g) | 1.00 (0.98, 1.01) 0.600 | 1.00 (0.98, 1.01) 0.600 | 1.00 (0.98, 1.01) 0.600 |
| Monounsaturated fatty acids (g) | 0.99 (0.97, 1.01) 0.300 | 0.99 (0.97, 1.01) 0.300 | 0.99 (0.97, 1.01) 0.300 |
| Polyunsaturated fatty acids (g) | 0.98 (0.95, 1.00) 0.094 | 0.98 (0.95, 1.01) 0.094 | 0.98 (0.95, 1.00) 0.094 |
| n-3 Fatty acids (g) | 0.89 (0.71, 1.12) 0.300 | 0.89 (0.71, 1.12) 0.300 | 0.89 (0.71, 1.12) 0.300 |
| n-6 Fatty acids (g) | 0.98 (0.95, 1.00) 0.082 | 0.98 (0.95, 1.01) 0.082 | 0.98 (0.95, 1.00) 0.082 |
| Energy (kcal) | 1.00 (1.00, 1.00) 0.200 | 1.00 (1.00, 1.00) 0.200 | 1.00 (1.00, 1.00) 0.200 |
| Protein (g) | 1.00 (0.98, 1.01) 0.400 | 1.00 (0.98, 1.01) 0.400 | 1.00 (0.98, 1.01) 0.235 |
| Carbohydrate (g) | 1.00 (1.00, 1.00) 0.092 | 1.00 (1.00, 1.00) 0.092 | 1.00 (1.00, 1.00) 0.092 |
| Cholesterol (mg) | 1.00 (1.00, 1.00) 0.900 | 1.00 (1.00, 1.00) 0.900 | 1.00 (1.00, 1.00) 0.900 |
| **Dietary fiber (g)** | **0.96 (0.92, 0.99) 0.019** | **0.96 (0.92, 0.98) 0.019** | **0.96 (0.92, 0.99) 0.019** |
| **Caffeine (mg)** | **4.33 (2.57, 7.28) <0.001** | **4.34 (2.59, 7.29) <0.001** | **4.31 (2.45, 7.49) <0.001** |
| Alcohol (g) | 1.01 (1.00, 1.01) 0.200 | 1.01 (1.00, 1.01) 0.200 | 1.01 (1.00, 1.01) 0.200 |
| Folic acid (mcg) | 1.00 (1.00, 1.00) 0.300 | 1.00 (1.00, 1.00) 0.300 | 1.00 (1.00, 1.00) 0.219 |
| Riboflavin (mg) | 0.98 (0.80, 1.19) 0.800 | 0.98 (0.80, 1.19) 0.800 | 0.98 (0.80, 1.19) 0.800 |
| β-Carotene (mcg) | 1.00 (1.00, 1.00) 0.600 | 1.00 (1.00, 1.00) 0.600 | 1.00 (1.00, 1.00) 0.600 |
| **Thiamin (mg)** | **0.65 (0.42, 1.00) 0.049** | **0.65 (0.41, 0.97) 0.049** | **0.71 (0.41, 0.99) 0.051** |
| Vitamin B6 (mg) | 1.07 (0.95, 1.20) 0.300 | 1.07 (0.95, 1.20) 0.300 | 1.07 (0.95, 1.20) 0.300 |
| Niacin (mg) | 1.00 (0.97, 1.03) 0.900 | 1.00 (0.97, 1.03) 0.900 | 1.00 (0.98, 1.03) 0.900 |
| Vitamin B12 (mcg) | 1.02 (0.99, 1.04) 0.150 | 1.02 (0.99, 1.04) 0.150 | 1.02 (0.99, 1.04) 0.150 |
| Vitamin C (mg) | 1.00 (0.99, 1.00) 0.200 | 1.00 (0.99, 1.00) 0.200 | 1.00 (0.98, 1.00) 0.200 |
| Vitamin D (mcg) | 0.99 (0.93, 1.06) 0.800 | 0.99 (0.93, 1.06) 0.800 | 0.99 (0.93, 1.06) 0.800 |
| Vitamin A (mcg) | 1.00 (1.00, 1.00) >0.9 | 1.00 (1.00, 1.00) >0.9 | 1.00 (1.00, 1.00) >0.9 |
| Magnesium (mg) | 1.00 (1.00, 1.00) 0.051 | 1.00 (1.00, 1.00) 0.057 | 1.00 (1.00, 1.00) 0.059 |
| Iron (mg) | 0.96 (0.92, 1.00) 0.071 | 0.96 (0.92, 1.00) 0.071 | 0.96 (0.93, 1.00) 0.071 |
| Zinc (mg) | 0.97 (0.91, 1.04) 0.400 | 0.97 (0.91, 1.03) 0.400 | 0.97 (0.91, 1.04) 0.345 |
| Selenium (mg) | 1.00 (0.99, 1.00) 0.500 | 1.00 (0.99, 1.00) 0.500 | 1.00 (0.99, 1.00) 0.501 |

Data are presented as OR [95% confidence interval] P-value.

Model 0, No covariate was adjusted.

Model Ⅰ, Age, race, marital status, and education level were adjusted.

Model II: Age, race, marital status, education level, PIR, exercise status, smoking status, alcohol consumption, BMI, hypertension, diabetes, use of female hormones, and use of birth control pills were adjusted.

Abbreviations: OR, Odds Ratio, PIR, Ratio of Family Income to Poverty; BMI, Body Mass Index
