# Supplementary figures and images for: Association of dietary inflammatory index with gynecological cancers in NHANES 2011–2018
Source: Front Nutr. 2025 May 12;12:1560987. doi: 10.3389/fnut.2025.1560987 (PMC12104050; doi:10.3389/fnut.2025.1560987)

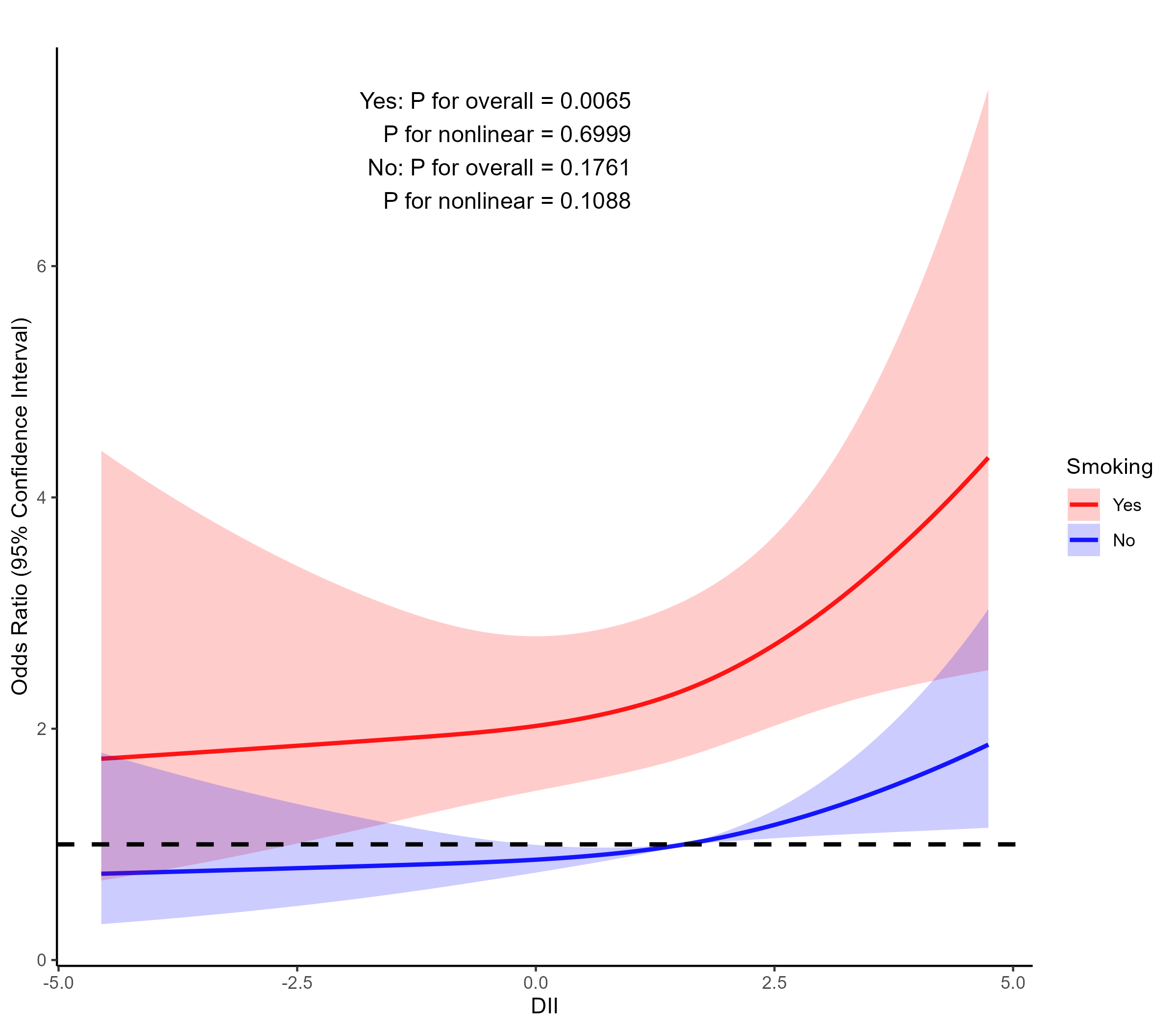

Supplement: Supplementary Figure 1 — The association between DII and gynecological cancers stratified by smoking status. [file Image_1.tiff]

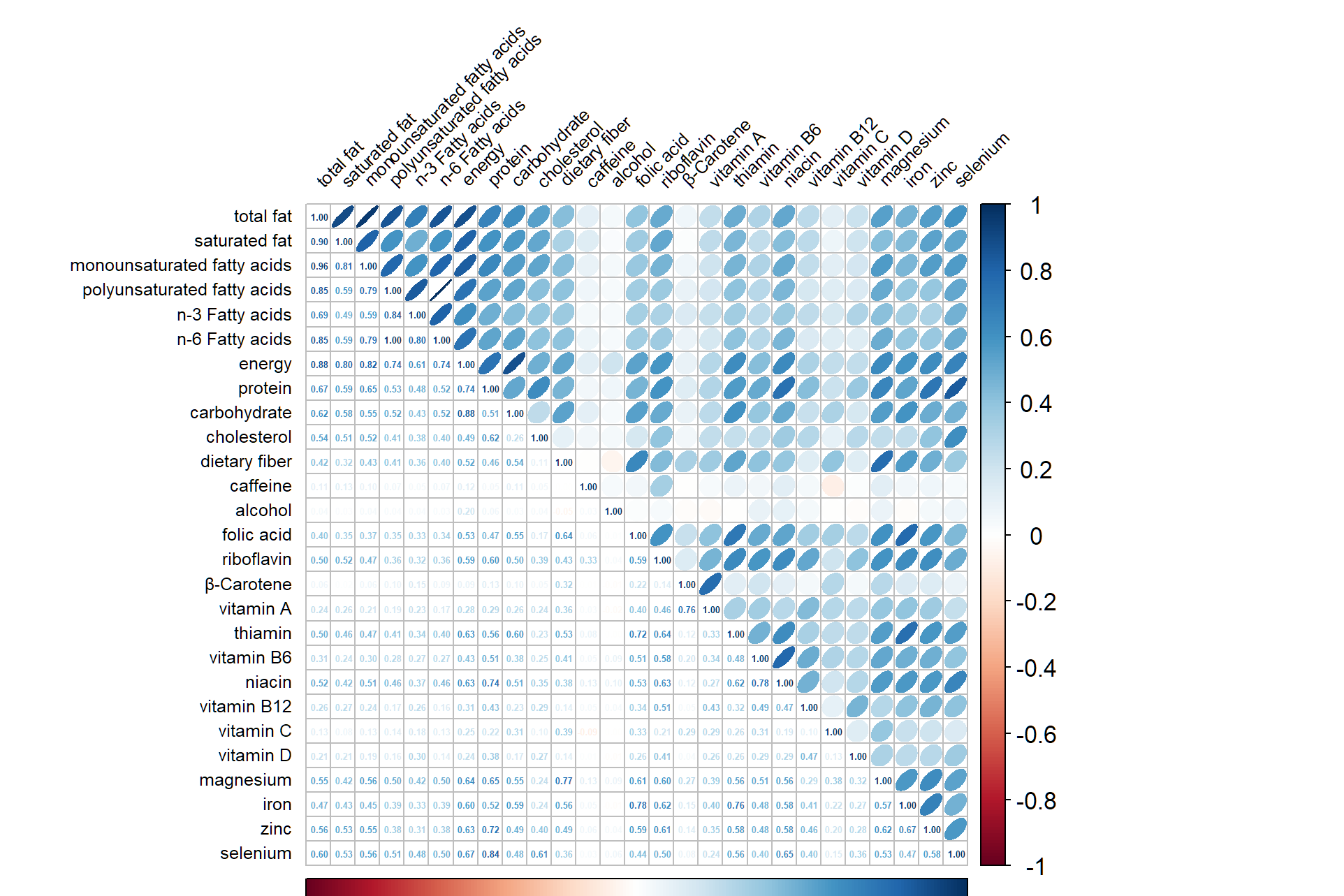

Supplement: Supplementary Figure 2 — Pearson correlations among the 27 dietary components. [file Image_2.tiff]

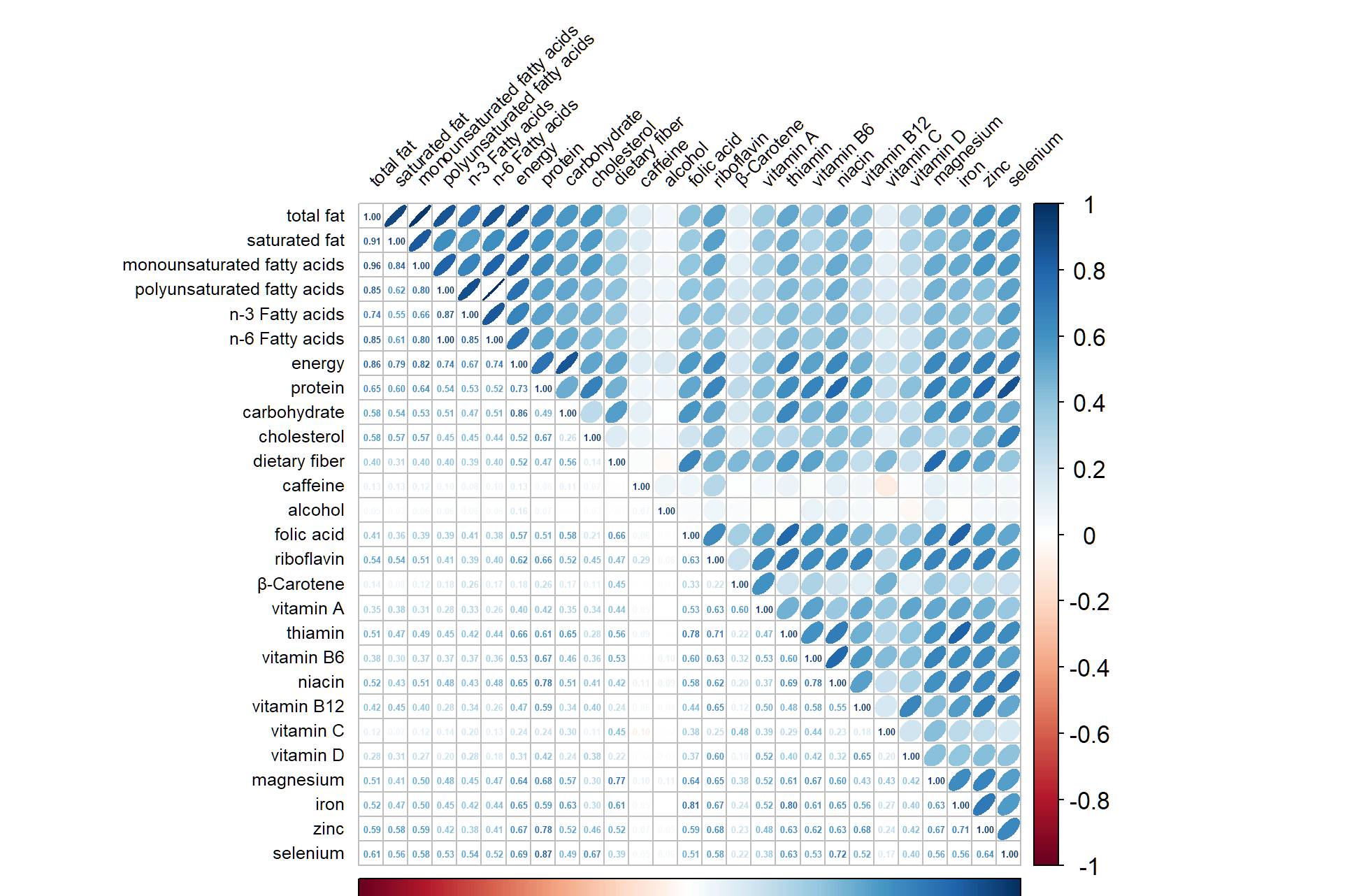

Supplement: Supplementary Figure 3 — Spearman correlations among the 27 dietary components. [file Image_3.tiff]
